# Enhanced Efficacy of Synbiotics Compared to Antibiotics in Promoting Growth, Intestinal Health, and Immune Response in Stinging Catfish

**DOI:** 10.1155/anu/2158993

**Published:** 2026-01-28

**Authors:** Md Nazmul Islam Nayan, Md. Saeduzzaman Faraji, Md. Zahid Hasan, Ibnath Haque Abony, Uruba Saiyara, M. Sadiqul Islam

**Affiliations:** ^1^ Department of Marine Fisheries Science, Bangladesh Agricultural University, Mymensingh, 2202, Bangladesh, bau.edu.bd; ^2^ Chattogram Medical College, Chattogram, 4202, Bangladesh

**Keywords:** aquaculture, digestive health, gut microbiota, *Heteropneustes fossilis*, synbiotic effects

## Abstract

This study evaluated the comparative efficacy of synbiotics, probiotics, prebiotics, and antibiotics in enhancing the growth performance, intestinal health, hepatic regeneration, and immune response of stinging catfish (*Heteropneustes fossilis*). Over a 45‐day experimental period, fingerlings were fed diets supplemented with antibiotics (Erisen‐Vet or Renamycin), probiotics (Everfresh‐Pro), prebiotics (onion powder), or a synbiotic combination (Everfresh‐Pro + onion powder). The results demonstrated that the *T*_Syn_ group consistently outperformed all other treatments. Quantitatively, the synbiotic group achieved the highest final weight (15.75 ± 1.77 g) and weight gain (11.75 ± 1.25 g) compared to the control (*T*_Con_) (4.01 ± 0.44 g weight gain). The specific growth rate (SGR) was significantly higher in *T*_Syn_ (3.05% day^−1^) than in antibiotic groups, while the feed conversion ratio (FCR) was optimized at 0.98 in *T*_Syn_ versus 1.35 in *T*_Con_. Intestinal histomorphology showed marked improvements in the *T*_Syn_ group, with villus length reaching 368.54 ± 21.52 µm and villus area at 85.93 ± 5.91 mm^2^, significantly exceeding both antibiotic and control values (*p* < 0.01). Immune response indicators were similarly enhanced; the total white blood cell (WBC) count in *T*_Syn_ rose to 11.26 × 10^3^/mm^3^ by day 45, compared to 8.45 × 10^3^/mm^3^ in the control. Furthermore, mucosal immunity was bolstered in the synbiotic group, which exhibited the highest goblet cell count (84.00 ± 3.00) and widest lamina propria (7.04 ± 0.81 µm). In conclusion, synbiotics not only promote superior growth and feed efficiency but also significantly enhance gut health, hepatic regeneration, and immune profiles. These findings provide strong evidence that synbiotics are a superior, sustainable alternative to antibiotics for the intensive culture of stinging catfish.

## 1. Introduction

Aquaculture has become one of the fastest‐growing sectors in global food production, playing a crucial role in meeting the rising demand for animal protein [1, 2]. However, intensification of aquaculture practices often leads to increased susceptibility of cultured species to stress, disease outbreaks, and poor growth performance. In response, antibiotics have long been employed in aquaculture systems to enhance growth and prevent infections [3]. While initially effective, the widespread and often indiscriminate use of antibiotics has raised serious concerns regarding the development of antimicrobial resistance, accumulation of harmful residues in aquatic ecosystems, and negative impacts on human and animal health [4–6].

To address these issues, the aquaculture industry is actively exploring sustainable and environmentally friendly alternatives to antibiotics [7]. Among the most promising solutions are synbiotics—a combination of probiotics (beneficial microorganisms) and prebiotics (nondigestible food ingredients that promote the growth of beneficial bacteria) [8–10]. Previous studies have shown that synbiotic supplementation significantly enhances growth performance, immune responses, and disease resistance in various fish species. For instance, synbiotics have been reported to improve weight gain in common carp [11], strengthen immune function and increase resistance to *Streptococcus iniae* in Asian seabass [12], and enhance immunity and survival rates (SRs) in sharptooth catfish challenged with *Aeromonas hydrophila* [13]. Additionally, synbiotic administration has been associated with elevated immune indicators, including increased white blood cell (WBC) counts [14]. Synbiotics function by enhancing the gut microbiota, boosting immune responses, and improving nutrient utilization [10, 15, 16]. These effects collectively contribute to better growth performance, disease resistance, and overall health in aquatic species [9, 17]. Unlike antibiotics, synbiotics do not pose a risk of resistance development or environmental contamination, making them an attractive option for sustainable aquaculture [18–22].

*Heteropneustes fossilis*, commonly known as the stinging catfish, is a commercially important freshwater fish species in South and Southeast Asia. It is valued for its high nutritional content, adaptability to varying environmental conditions, and consistently high demand in local and global markets [10]. Despite its resilience, optimal production of *H. fossilis* is often hindered by poor feed efficiency, disease susceptibility, and water quality issues—factors that are typically addressed through antibiotic use. As sustainability becomes a priority, there is a critical need to evaluate alternative strategies, such as synbiotics, that can enhance the productivity and health of this species without adverse ecological consequences [5].

Although probiotics, prebiotics, and synbiotics have been widely studied in aquaculture, clear evidence directly comparing their combined effects with commonly used antibiotics across multiple physiological systems remains limited, particularly for *H. fossilis*. The innovation of the present study lies in its comprehensive, side‐by‐side evaluation of synbiotics versus conventional antibiotics by simultaneously integrating growth performance, detailed intestinal histomorphology, hepatic cellular regeneration, and both quantitative and qualitative immune responses. Unlike many previous studies that focused on single health indicators or excluded antibiotic benchmarks, this work provides a holistic assessment of functional feed additives under controlled conditions using commercially relevant antibiotics. Moreover, the use of a natural prebiotic source (onion powder) combined with a multistrain probiotic offers a practical and scalable synbiotic formulation for aquaculture. By demonstrating that synbiotics not only replace but also consistently outperform antibiotics across all measured parameters, this study advances current knowledge and provides novel, species‐specific evidence supporting synbiotics as a superior and sustainable alternative for stinging catfish culture.

## 2. Materials and Methods

The experiment was conducted in the wet laboratory—equipped with water supply, ventilation, and specialized utilities for handling biological materials—of the Department of Marine Fisheries Science, Bangladesh Agricultural University. A total of 18 plastic tanks (60 × 45 × 35 cm) with a 90‐L capacity were utilized for the study. Before use, the tanks underwent a thorough disinfection with a potassium permanganate solution, followed by rinsing with tap water and subsequent air‐drying. The experimental design was completely randomized, consisting of six treatment groups, each with three replicates.

Fingerlings of stinging catfish (*Heteropneustes fossilis*), with an average initial weight of 3.98 ± 0.26 g (mean ± SD), were sourced from a local hatchery. They were acclimatized in polybags for 3 h, followed by a 5‐min 0.3% salt bath treatment to reduce external pathogens, and kept in aerated tanks for 1 week before the experiment began. Fish were stocked in the plastic tanks at a density of 2500 fish per decimal. The experiment was conducted over a period of 45 days, with growth performance parameters evaluated at 15‐day intervals. A daily water exchange of 10% was performed in each tank, and continuous aeration was maintained throughout the experimental period. All experimental procedures were conducted in accordance with the guidelines of the Animal Care Committee, Bangladesh Agricultural University, Bangladesh.

Three feed additives were used: probiotics, prebiotics, and synbiotics. The probiotic was a commercial product, Everfresh‐Pro, containing a mixture of *Bacillus subtilis*, *B. licheniformis*, *B. megaterium*, and *B. pumilus*. Farmer’s Gold dried onion (*Allium cepa*) powder, a natural source of prebiotics including fructooligosaccharides (FOS) and inulin [23], was purchased from an authorized local dealer (Organic Hat Bazar, Bangladesh). Two broad‐spectrum antibiotics were incorporated into the experimental diets: Erisen‐Vet, which contains erythromycin, sulfadiazine, and trimethoprim, and Renamycin, which contains oxytetracycline. Each antibiotic was added at a rate of 5 g/kg to the basal diet. The basal diet itself was a commercial pelleted feed (Spectra Fish Feed Ltd.) with a crude protein content of 37%. Six experimental treatment groups were prepared. The control group (*T*_Con_) received only the basal diet. The first antibiotic group (*T*_Ant1_) was fed the basal diet supplemented with Erisen‐Vet at 5 g/kg, while the second antibiotic group (*T*_Ant2_) received the basal diet with Renamycin at the same concentration. The probiotic group (*T*_Pro_) was given the basal diet supplemented with 40 g/kg of Everfresh‐Pro. The prebiotic group (*T*_Pre_) received the basal diet with 20 g/kg of onion powder. Finally, the synbiotic group (*T*_Syn_) was provided a combination of Everfresh‐Pro (40 g/kg) and onion powder (20 g/kg) mixed into the basal diet. All diets were thoroughly mixed, air‐dried at room temperature for 24 h, and stored in sealed containers under refrigeration. Fish were fed twice daily at a rate equivalent to 5% of their body weight.

In each tank, a minimum of five fish were randomly selected for individual weight measurements. SRs were recorded for each replicate tank, and daily feed intake was monitored throughout the experimental period. Growth performance parameters were calculated using the formulas described by Kari et al. [24].

1. Weight gain (g) = Mean final weight − Mean initial weight
2. $\text{Percentage}\;\text{of}\;\text{weight}\;\text{gain}\;\left(\%\right)=\frac{\left(\text{Mean}\;\text{final}\;\text{weight }-\;\text{Mean}\;\text{initial}\;\text{weight}\right)}{\text{Mean}\;\text{initial}\;\text{weight}}\times 100$
3. $\text{Average}\;\text{daily}\;\text{weight}\;\text{gain}\;\left({\text{g day}}^{-1}\right)=\frac{\left(\text{Mean}\;\text{final}\;\text{weight }-\text{Mean}\;\text{initial}\;\text{weight}\right)}{\text{Days}}$
4. $\text{Specific}\;\text{growth}\;\text{rate}\;\left(\%\;{\text{day}}^{-1}\right)=\frac{\left(\ln\;\text{final}\;\text{weight}\;-\ln\;\text{initial}\;\text{weight}\right)}{\text{Days}}\times 100$
5. $\text{Feed}\;\text{conversion}\;\text{ratio}\;\left(\text{FCR}\right)=\frac{\text{Feed}\;\text{fed}\;\left(\text{dry}\;\text{weight}\right)}{\text{Live}\;\text{weight}\;\text{gain}\;\left(\text{g}\right)}$
6. $\text{Hepatosomatic}\;\text{index}\;\left(\text{HSI}\%\right)=\frac{\text{Liver}\;\text{weight}\;\left(\text{g}\right)}{\text{Total}\;\text{fish}\;\text{weight}\;\left(\text{g}\right)}\times 100$
7. $\text{Viscerasomatic}\;\text{index}\;\left(\text{VSI}\%\right)=\frac{\text{Viscera}\;\text{weight}\;\left(\text{g}\right)}{\text{Total}\;\text{fish}\;\text{weight}\;\left(\text{g}\right)}\times 100$
8. $\text{Survival}\;\text{rate}\;\left(\text{SR}\%\right)=\frac{\text{Number}\;\text{of fish harvested}}{\text{Number of fish stocked at the start of the experiment}}\times 100$

For hematological analysis, blood samples were collected from five fish on each sampling day via the caudal vein using sterile tips, following the method described by Islam et al. [25]. To minimize stress‐related variations, blood was withdrawn within 1 min per fish. The samples were mixed with WBC dilution fluid and N/10 hydrochloric acid for WBC estimation. WBC counts (×10^3^ cells/mm^3^) were performed using an Improved Neubauer hemocytometer [26], with 10 µL of diluted blood placed under a cover slip and viewed at 40× magnification. Cells were counted and calculated using Zhang et al.’s [27] formulae. For differential WBC counts, blood smears were air‐dried and stained with Leishman stain. Two hundred cells were counted manually and classified based on morphology, as described by Blumenreich [28]. While automated counters exist, manual methods are preferred for their accuracy in identifying morphological abnormalities. Essential hematological parameters like total and differential WBC counts were recorded.

Histomorphological analysis of the liver and intestine was performed on three fish sampled per replicate at 15‐day intervals. The fish were euthanized according to the methods of Islam et al. [21] and [22]. Subsequently, liver and intestinal tissues were fixed in 10% formalin and processed via dehydration, clearing, and paraffin embedding. Tissue sections (5 µm) were cut using a microtome, stained with hematoxylin and eosin, and mounted with DPX. Slides were examined under an Optika B‐190 microscope with ToupView software. Morphological parameters, including villus height, width, area, crypt depth, and muscle thickness, were measured. Villus surface area was calculated using the formula: 2π × (villus width/2) × height.

Statistical analysis was performed using Microsoft Excel 2016, SPSS, and Statistics 10. One‐way ANOVA was used to determine significant differences (*p* < 0.05), followed by Tukey’s post hoc test for multiple comparisons.

## 3. Results

### 3.1. Comparative Effects of Synbiotics and Antibiotics on the Growth Performances of Stinging Catfish

The growth performance of stinging catfish significantly varied across the treatment groups over the 45‐day trial (Table 1). While initial weights were comparable among all groups (*p*  > 0.05), final weight, weight gain, percentage weight gain (%WG), specific growth rate (SGR), and feed conversion ratio (FCR) differed significantly (*p*  < 0.01). The highest final weight (15.75 ± 1.77 g) and weight gain (11.75 ± 1.25 g) were recorded in the synbiotic‐treated group (*T*_Syn_), followed by the probiotic (*T*_Pro_) and prebiotic (*T*_Pre_) groups. These groups also exhibited significantly higher %WG and SGR, with *T*_Syn_ achieving the highest values (294.08% and 3.05%.day^−1^, respectively). Fish treated with antibiotics (*T*_Ant1_ and *T*_Ant2_) showed improvement in growth performance compared to the control (*T*_Con_), but their values remained significantly lower than those of the synbiotic and probiotic groups (*p*  < 0.05). The FCR was lowest in *T*_Syn_ (0.98), indicating more efficient feed utilization, while *T*_Con_ showed the highest FCR (1.35). No significant differences were observed among treatments for hepatosomatic index (HSI) and viscerosomatic index (VSI) (*p*  > 0.05), suggesting that liver and visceral organ development were not adversely affected by the treatments.

**Table 1:** Growth performance of stinging catfish treated with synbiotics and antibiotics over 45 days.

Treatment	Initial weight (g)	Final weight (g)	W. gain (g)	% W. gain	SGR (%.day^−1^)	FCR	HSI %	VSI %
*T*_Con_	3.97 ± 0.02	7.40 ± 0.57^d^	3.43 ± 0.4^d^	86.40^d^	1.38^d^	1.35	0.77	1.32
*T*_Ant1_	3.97 ± 0.01	9.67 ± 0.24^c^	5.71 ± 0.1^c^	144.44^c^	1.99^c^	1.22	1.03	1.52
*T*_Ant2_	3.99 ± 0.03	8.57 ± 0.20^cd^	4.58 ± 0.17^cd^	114.82^cd^	1.70^cd^	1.27	1.12	1.05
*T*_Pre_	4.00 ± 0.01	11.50 ± 2.83^b^	8.15 ± 0.14^b^	188.22^bc^	2.35^b^	1.18	0.58	0.81
*T*_Pro_	3.99 ± 0.03	12.14 ± 0.20^b^	7.51 ± 1.97^b^	203.82^b^	2.47^b^	1.09	1.01	1.53
*T*_Syn_	4.00 ± 0.01	15.75 ± 1.77^a^	11.75 ± 1.25^a^	294.08^a^	3.05^a^	0.98	1.33	1.64
*p*‐Value	0.114	0.001	0.001	0.001	0.001	0.001	0.301	0.518
Significant level	NS	^∗∗^	^∗∗^	^∗∗^	^∗∗^	^∗∗^	NS	NS

^∗∗^Indicates highly significant at a 5% level of probability. According to Turkey’s HSD test in a column, values with the same letter do not differ significantly (*p*  < 0.05), whereas those with dissimilar letters differ significantly.

**Figure 1:**
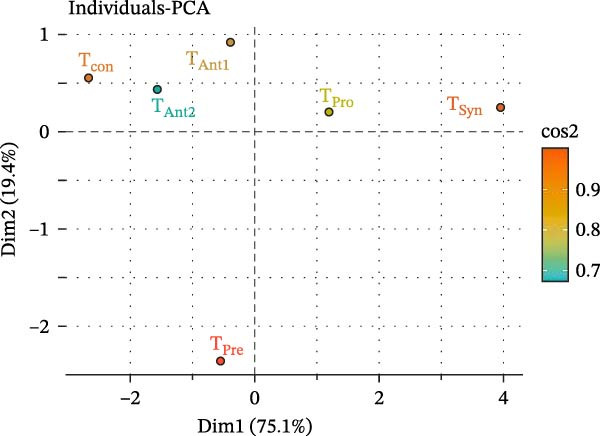
Principal component analysis (PCA) plot showing the clustering of different treatments (*T*_Con_, *T*_Ant1_, *T*_Ant2_, *T*_Pre_, *T*_Pro_, and *T*_Syn_) based on their multivariate effects on measured parameters in stinging catfish.

Principal component analysis (PCA) was conducted to explore the variance in growth performance parameters among treatment groups. The first two principal components (Dim1 and Dim2) explained a cumulative 94.5% of the total variance, with Dim1 accounting for 75.1% and Dim2 accounting for 19.4% (Figure 1). The PCA biplot revealed clear clustering and separation among treatments. Notably, *T*_Syn_ was positioned farthest along the positive axis of Dim1, suggesting a strong influence on the primary source of variance in growth performance. *T*_Pre_ appeared in the negative region of both dimensions, indicating distinct growth responses compared to other treatments. Antibiotic treatments (*T*_Ant1_ and *T*_Ant2_) clustered closer to the center, implying moderate and similar effects. *T*_Con_ (control) was separated from synbiotic and probiotic treatments, indicating a lesser impact on performance indicators. The quality of representation (cos2 values) indicated that *T*_Syn_ and *T*_Pre_ had high contributions to the principal component axes (cos2 > 0.9), supporting their significance in differentiating growth outcomes among treatments.

### 3.2. Comparative Effects of Synbiotics and Antibiotics on the Intestinal Histomorphology

Intestinal morphology parameters of stinging catfish were significantly influenced by dietary treatments over the 45‐day period (Table 2). The synbiotic‐treated group (*T*_Syn_) exhibited the most pronounced improvements across all measured parameters. Villus length, width, and area were significantly higher in *T*_Syn_ (368.54 ± 21.52 mm^2^, 74.22 ± 3.25 mm^2^, and 85.93 ± 5.91 mm^2^, respectively) compared to all other groups (*p*  < 0.01), indicating enhanced absorptive surface area. Probiotic (*T*_Pro_) and prebiotic (*T*_Pre_) treatments also significantly increased villus metrics compared to the control (*T*_Con_), though to a lesser extent than *T*_Syn_. Antibiotic‐treated groups (*T*_Ant1_ and *T*_Ant2_) showed moderate improvements but were still significantly lower than the synbiotic and probiotic groups in most parameters (*p*  < 0.05). Crypt depth was significantly higher in *T*_Syn_ (39.25 ± 0.97 µm), suggesting enhanced cell proliferation activity, while the lowest depth was observed in *T*_Con_ (27.54 ± 0.65 µm). Similarly, wall thickness and muscular layer thickness were significantly greater in *T*_Syn_ (9.93 ± 0.65 µm and 36.42 ± 1.63 µm, respectively), indicating improved structural development and possibly enhanced gut motility and nutrient absorption capacity.

**Table 2:** Alterations in intestinal morphology of stinging catfish treated with synbiotics and antibiotics over 45 days.

Treatment	V. length (mm^2^)	V. width (mm^2^)	V. area (mm^2^)	Crypt depth (µm)	Thickness of wall (µm)	Thickness of muscular (µm)
*T*_Con_	225.54 ± 27.33^d^	51.04 ± 5.01^b^	36.17 ± 3.11^c^	27.54 ± 0.65^b^	4.03 ± 0.87^b^	19.04 ± 2.23^c^
*T*_Ant1_	266.04 ± 18.60^c^	55.64 ± 9.80^b^	46.50 ± 8.93^bc^	31.56 ± 3.30^ab^	5.57 ± 0.93^b^	25.18 ± 1.83^bc^
*T*_Ant2_	245.81 ± 12.02^cd^	51.04 ± 4.58^b^	39.41 ± 2.29^c^	29.36 ± 0.57^ab^	4.93 ± 1.60^b^	23.46 ± 2.77^bc^
*T*_Pre_	292.86 ± 24.69^b^	58.31 ± 1.41^b^	53.65 ± 5.55^bc^	32.92 ± 3.35^ab^	7.32 ± 1.88^ab^	29.82 ± 5.26^b^
*T*_Pro_	332.57 ± 54.50^ab^	63.71 ± 3.42^ab^	66.56 ± 7.71^b^	34.19 ± 7.73^ab^	6.27 ± 0.95^b^	28.28 ± 2.47^b^
*T*_Syn_	368.54 ± 21.52^a^	74.22 ± 3.25^a^	85.93 ± 5.91^a^	39.25 ± 0.97^a^	9.93 ± 0.65^a^	36.42 ± 1.63^a^

*p*‐Value	0.001	0.001	0.001	0.032	0.001	0.001

Significant level	^∗∗^	^∗∗^	^∗∗^	^∗^	^∗∗^	^∗∗^

^∗^Indicates statistical significance.

^∗∗^Indicates high statistical significance at the 5% probability level. According to Tukey’s HSD test, values within a column that share the same letter are not significantly different (*p*  < 0.05), whereas values with different letters differ significantly.

### 3.3. Comparative Effects of Synbiotics and Antibiotics on the Hepatic Cells

**Figure 2:**
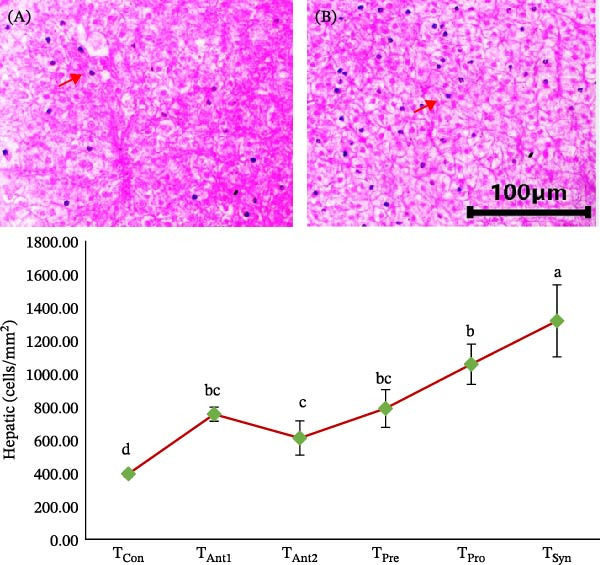
Comparative effects of various treatments on hepatic cell density (cells/mm^2^) in the liver of stinging catfish. Values with different alphabet superscripts differ significantly (*p*  < 0.05). All values expressed as mean ± SD. Hepatic cells are indicated by the arrows in both photomicrographs, comparing *T*_Ant1_ (A) with *T*_Syn_ (B).

The hepatic cell density in the liver of stinging catfish varied significantly among the treatment groups (Figure 2). The highest hepatic cell density was observed in the *T*_Syn_ group, followed by *T*_Pro_, both of which showed significantly higher values compared to the control (*T*_Con_) and antibiotic‐treated groups (*T*_Ant1_ and *T*_Ant2_) (*p*  < 0.05). *T*_Con_ exhibited the lowest cell density, while *T*_Ant1_ and *T*_Ant2_ showed moderate increases but remained significantly lower than the synbiotic and probiotic groups. No significant difference was observed between *T*_Ant1_, *T*_Ant2_, and *T*_Pre_. Treatments with prebiotics (*T*_Pre_) showed intermediate results, not significantly different from the antibiotic groups.

### 3.4. Comparative Effects of Synbiotics and Antibiotics on the WBC Cell Counts

The WBC counts of stinging catfish varied significantly (*p*  < 0.05) across treatments at both 30 and 45 days (Table 3). At day 30, the highest WBC count was recorded in the synbiotic‐treated group (*T*_Syn_) at 8.10 × 10^3^/mm^3^, which was significantly higher than all other groups (*p* < 0.05). The control group (*T*_Con_) exhibited the lowest WBC count (7.30 × 10^3^/mm^3^), indicating reduced immune cell activity in the absence of functional feed additives. By day 45, the *T*_Syn_ group again demonstrated the most substantial immune response, with a WBC count of 11.26 × 10^3^/mm^3^, significantly higher than all other treatments (*p*  < 0.05). The probiotic (*T*_Pro_) group also showed a marked increase in WBC levels (10.31 × 10^3^/mm^3^), followed by the prebiotic (*T*_Pre_) and antibiotic‐treated groups (*T*_Ant1_ and *T*_Ant2_). The control group remained the lowest (8.45 × 10^3^/mm^3^), with minimal improvement over time.

**Table 3:** Comparative effects of synbiotics and antibiotics on WBC cell counts of stinging catfish at different time intervals.

Treatment	White blood cell (×10^3^/mm^3^)	
	30 days	45 days
*T*_Con_	7.30 ± 0.29^c^	8.45 ± 0.05^d^
*T*_Anti1_	7.63 ± 0.03^b^	9.88 ± 0.20^c^
*T*_Anti2_	7.51 ± 0.10^b^	9.21 ± 0.07^cd^
*T*_Pre_	7.48 ± 0.09^bc^	9.96 ± 0.10^c^
*T*_Pro_	7.61 ± 0.14^b^	10.31 ± 0.01^b^
*T*_Syn_	8.10 ± 0.02^a^	11.26 ± 0.06^a^
*p*‐Value	0.001	0.001
Level of significance	^∗^	^∗^

*Note:* Values are presented as mean ± standard deviation (SD). Different superscript letters in the same column indicate a significant difference (*p*  < 0.05).

### 3.5. Comparative Effects of Synbiotics and Antibiotics on the Immune Response Indicators

**Figure 3:**
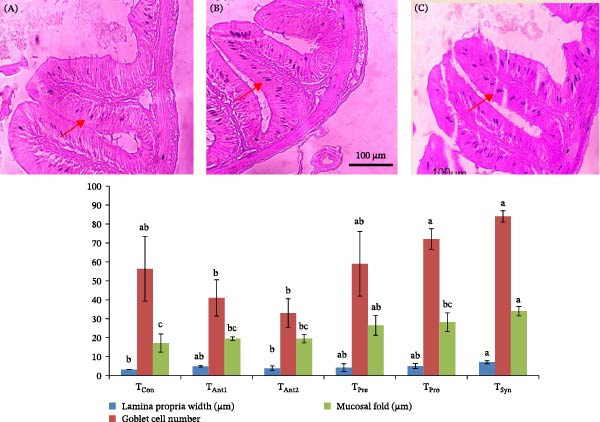
Effects of synbiotics and antibiotics on immune response indicators in the intestinal histology of stinging catfish. Values with different alphabet superscripts differ significantly (*p*  < 0.05). All values expressed as mean ± SD. Goblet cells, indicated by the arrows in the photomicrographs, are compared among *T*_Ant1_ (A), *T*_Pro_ (B), and T_Syn_ (C).

Histological examination of intestinal sections showed that different dietary treatments had a significant impact on key immune response indicators in stinging catfish, as shown in Figure 3. Synbiotic supplementation (*T*_Syn_) resulted in the most pronounced enhancements across all measured parameters, indicating a stronger intestinal immune response. Lamina propria width was significantly higher in *T*_Syn_ (7.04 ± 0.81 µm) compared to all other groups (*p*  < 0.05), suggesting enhanced infiltration of immune cells and increased mucosal immunity. Probiotic (*T*_Pro_) and prebiotic (*T*_Pre_) treatments also increased lamina propria width, although not significantly different from the antibiotic groups (*T*_Ant1_ and *T*_Ant2_). Goblet cell numbers, essential for mucin production and mucosal barrier integrity, were highest in the *T*_Syn_ group (84.00 ± 3.00), followed by *T*_Pro_ (72.33 ± 5.51), both significantly higher than antibiotic‐treated and control groups (*p*  < 0.01). Antibiotic treatments (*T*_Ant1_ and *T*_Ant2_) showed the lowest goblet cell counts, indicating a suppressed mucosal immune response. Mucosal fold height was also significantly elevated in the *T*_Syn_ group (34.01 ± 2.47 µm), suggesting increased absorptive surface area and improved intestinal function. Probiotic and prebiotic groups showed moderate increases, while *T*_Con_ and antibiotic groups exhibited significantly shorter mucosal folds (*p*  < 0.01).

### 3.6. Comparative Effects of Synbiotics and Antibiotics on the Differential WBC Counts

**Figure 4:**
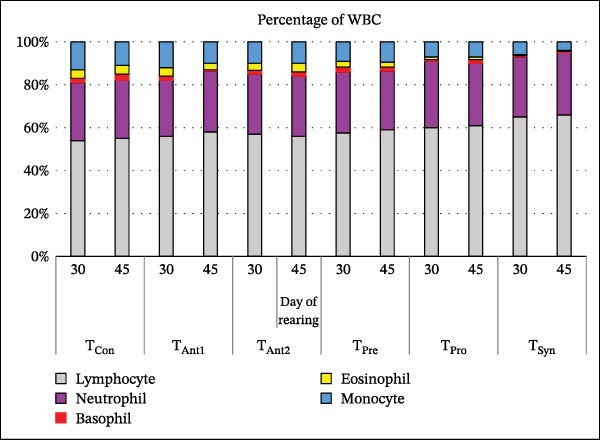
Differential WBC counts in stinging catfish after synbiotic and antibiotic treatments.

Differential WBC counts revealed notable changes in immune cell composition among treatment groups at both 30 and 45 days (Figure 4). Across all treatments, lymphocytes and neutrophils represented the most abundant WBC types. Among the groups, the *T*_Syn_ group demonstrated the most favorable immune profile, with the highest percentage of lymphocytes and a notably lower proportion of neutrophils, indicating a stronger adaptive immune response and reduced inflammatory activity. In contrast, antibiotic‐treated groups showed lower lymphocyte levels and elevated neutrophil counts, suggesting a proinflammatory response. The *T*_Pre_ and *T*_Pro_ groups exhibited intermediate values, with modest increases in lymphocytes over time but still inferior to the *T*_Syn_ group. Monocyte, eosinophil, and basophil percentages remained relatively low and stable across all groups.

## 4. Discussion

This study evaluated the effects of synbiotics, probiotics, prebiotics, and antibiotics on the growth, intestinal health, liver regeneration, and immune response of stinging catfish. Synbiotic supplementation consistently outperformed all other treatments, including antibiotics, across key parameters. The *T*_Syn_‐fed fish showed significantly higher growth rates, better feed efficiency, and improved intestinal morphology, with increased villus dimensions. Immune responses were also enhanced, as reflected by elevated WBC counts, increased goblet cell numbers, and a favorable lymphocyte‐to‐neutrophil ratio. These findings highlight synbiotics as a promising, sustainable alternative to antibiotics in aquaculture.

Synbiotic supplementation led to significant improvements in key growth parameters of stinging catfish, including final weight, weight gain, %WG, SGR, and FCR. These enhancements align with previous findings in other aquaculture species where probiotic and prebiotic combinations produced synergistic effects [29, 30]. The *T*_Syn_ group achieved the highest final weight (15.75 g) and the lowest FCR (0.98), indicating improved nutrient assimilation and feed efficiency, consistent with observations in fish [22, 31–33]. Although both probiotics and prebiotics individually enhanced growth compared to the control group, their combined application produced a significantly greater effect. This finding supports the synergistic mechanism proposed by Gibson and Roberfroid [34] and further reinforced by subsequent studies (e.g., [35, 36]). Antibiotic‐treated groups (*T*_Ant1_ and *T*_Ant2_) showed only moderate gains and were clearly less effective than synbiotics. Additionally, the absence of significant differences in HSI and VSI across treatments suggests that growth was balanced and not linked to abnormal organ development.

Our histological analysis of the stinging catfish intestine demonstrates profound morphological improvements in response to the synbiotic treatment. The significant increases in villus length, width, and area, as well as crypt depth and muscular layer thickness, indicate a more developed and robust intestinal architecture. These structural enhancements are crucial for maximizing nutrient absorption and strengthening the gut barrier function, which are foundational for overall fish health and disease resistance. The superiority of the *T*_Syn_ group over its individual *T*_Pro_ and *T*_Pre_ components highlights a powerful synergistic effect. This is consistent with existing literature, which suggests that prebiotics act as a selective substrate for probiotics, promoting their survival and colonization in the host’s gut and leading to a more pronounced health benefit [9, 37–39]. Our findings provide strong evidence that this combined approach is more effective than single‐component supplementation. Furthermore, the marginal improvements observed in the antibiotic groups reinforce concerns about their limited efficacy and the need for sustainable alternatives in aquaculture. The synbiotic treatment provides a nonantimicrobial strategy that not only promotes intestinal health but also supports overall well‐being. This study underscores the potential of synbiotics as a key functional feed additive for enhancing gut integrity, improving digestive function, and reducing reliance on conventional antibiotic use in aquaculture [5, 20, 40, 41].

The significant increase in hepatic cell numbers in the *T*_Syn_ group suggests a pronounced hepatoprotective and regenerative effect in stinging catfish. Synbiotics are known to improve liver health by modulating gut microbiota and reducing systemic inflammation, which supports liver cell regeneration [37, 42, 43]. The *T*_Syn_ group’s superior hepatic cell density aligns with previous studies reporting enhanced liver function and morphology in fish fed synbiotic diets [18, 44–46]. Although *T*_Pro_ and *T*_Pre_ groups also improved hepatic cell numbers, their effects were less pronounced, highlighting the synergistic advantage of combining both components. Antibiotic treatments showed moderate or minimal improvements, possibly due to their limited impact on gut–liver axis regulation. These findings support synbiotics as a more effective alternative to antibiotics in promoting hepatic health and cellular regeneration in aquaculture species [47, 48].

The marked increase in WBC counts in the TSyn group at both 30 and 45 days suggests a potent immunostimulatory effect of synbiotics in stinging catfish [49]. Blood samples were collected from each fish in under 1 min, minimizing the likelihood of stress‐induced elevation in WBC levels. However, it is important to note that higher WBC counts do not always indicate stronger immunity, as they can also reflect infection, inflammation, or stress. Since bacterial interactions were not assessed in this study, our interpretation is necessarily limited. As highlighted by Lopez‐Gil et al. [50] and Kabat et al. [51], the coordination and function of immune cells are as important as their absolute numbers. Importantly, the WBC counts observed in this study (4.5–11 × 10^9^/L) fall within the normal range reported for healthy adults [52], supporting the reliability of our results and indicating no signs of illness. The *T*_Syn_ group consistently outperformed all other treatments, including antibiotics, probiotics, and prebiotics, aligning with previous findings that synbiotics synergistically enhance host immunity [53, 54]. Notably, the WBC count in the *T*_Syn_ group increased from 8.10 × 10^3^/mm^3^ to 11.26 × 10^3^/mm^3^ over 15 days, highlighting a time‐dependent immunomodulatory effect. While antibiotics did increase WBC counts compared to the control, they were less effective than synbiotics, reinforcing concerns over their long‐term use and resistance risks. These results support the use of synbiotics as a safer, more effective alternative for immune enhancement in aquaculture.

The findings of this study demonstrate that the synbiotic diet significantly enhances key indicators of intestinal health and immune response in stinging catfish. Histological analysis revealed a notable increase in lamina propria width, goblet cell count, and mucosal fold height in the *T*_Syn_ group compared to both antibiotic and control treatments. A wider lamina propria, rich in immune cells, is associated with a more robust innate immune system, capable of responding effectively to pathogens [10, 44, 55]. Furthermore, the increased density of goblet cells and taller mucosal folds are critical for maintaining gut barrier integrity. Goblet cells produce mucus that acts as a physical and chemical barrier against harmful microorganisms, a function that is enhanced with increased goblet cell numbers [56, 57]. Similarly, greater mucosal fold height expands the intestinal surface area, facilitating nutrient absorption and creating a more formidable defense against enteric pathogens [18, 39, 58]. These results highlight the potential of synbiotics as a superior and more sustainable alternative to antibiotics for improving fish health, growth, and disease resistance [16, 20, 21].

Synbiotic supplementation significantly influenced the differential WBC profile of stinging catfish, particularly in contrast to antibiotic treatments. Hematological parameters are reliable indicators of immune function and overall health status in fish. As shown in Figure 4, the synbiotic‐fed group exhibited a higher percentage of lymphocytes, the cornerstone of adaptive immunity, than all other treatment groups. This finding aligns with previous studies showing that synbiotics can stimulate the immune response in aquatic species [29, 59]. Conversely, the antibiotic groups (*T*_Anti1_ and *T*_Anti2_) showed an elevated percentage of neutrophils, a key indicator of inflammation or stress. This suggests that synbiotic supplementation can lead to a more balanced and robust immune system, offering a promising, sustainable alternative to traditional antibiotic use in aquaculture [60]. The stable percentages of other WBC types, such as monocytes and eosinophils, across all groups further support the conclusion that synbiotics effectively promote a state of health rather than a state of heightened immune activity in response to a pathogen.

## 5. Conclusion

This study conclusively demonstrates that synbiotics significantly enhance growth performance, intestinal health, hepatic cell regeneration, and immune response in stinging catfish. The synbiotic diet consistently outperformed antibiotics and other treatments, presenting a robust, sustainable alternative for aquaculture. By improving key physiological and immunological parameters, synbiotics offer a promising strategy to bolster fish health and productivity, mitigating the environmental and antimicrobial resistance concerns associated with conventional antibiotic use.

## Author Contributions

**Md Nazmul Islam Nayan:** writing – original draft, methodology, visualization. **Md. Saeduzzaman Faraji:** writing – original draft, visualization. **Md. Zahid Hasan:** methodology, visualization. **Ibnath Haque Abony and Uruba Saiyara:** review and editing. **M. Sadiqul Islam:** supervision, resources, conceptualization, review and editing, project administration.

## Funding

This work was funded by the Bangladesh Agricultural University Research System (BAURES), Bangladesh Agricultural University, Mymensingh 2202, Bangladesh.

## Disclosure

The authors are solely responsible for the content and writing of this paper.

## Ethics Statement

Experimental fish were handled according to the guidelines of the Animal Care Committee of Bangladesh Agricultural University, Bangladesh.

## Conflicts of Interest

The authors declare no conflicts of interest.

## Data Availability

The data that support the findings of this study are available from the corresponding author upon reasonable request.
